# Proteomic Analysis of the Relationship between Metabolism and Nonhost Resistance in Soybean Exposed to *Bipolaris maydis*

**DOI:** 10.1371/journal.pone.0141264

**Published:** 2015-10-29

**Authors:** Yumei Dong, Yuan Su, Ping Yu, Min Yang, Shusheng Zhu, Xinyue Mei, Xiahong He, Manhua Pan, Youyong Zhu, Chengyun Li

**Affiliations:** 1 Key Laboratory of Agro-Biodiversity and Pest Management of Education Ministry of China, Yunnan Agricultural University, Kunming, 650201, China; 2 The Life Science and Technology Department of Kunming University, Kunming, 650214, China; 3 Institute of Biotechnology and Germplasm Resources, Yunnan Academy of Agricultural Sciences, Kunming 650223, China; Henan Agricultural Univerisity, CHINA

## Abstract

Nonhost resistance (NHR) pertains to the most common form of plant resistance against pathogenic microorganisms of other species. *Bipolaris maydis* is a non-adapted pathogen affecting soybeans, particularly of maize/soybean intercropping systems. However, no experimental evidence has described the immune response of soybeans against *B*. *maydis*. To elucidate the molecular mechanism underlying NHR in soybeans, proteomics analysis based on two-dimensional polyacrylamide gel electrophoresis (2-DE) was performed to identify proteins involved in the soybean response to *B*. *maydis*. The spread of *B*. *maydis* spores across soybean leaves induced NHR throughout the plant, which mobilized almost all organelles and various metabolic processes in response to *B*. *maydis*. Some enzymes, including ribulose-1,5-bisphosphate carboxylase/oxygenase (RuBisCO), mitochondrial processing peptidase (MPP), oxygen evolving enhancer (OEE), and nucleoside diphosphate kinase (NDKs), were found to be related to NHR in soybeans. These enzymes have been identified in previous studies, and STRING analysis showed that most of the protein functions related to major metabolic processes were induced as a response to *B*. *maydis*, which suggested an array of complex interactions between soybeans and *B*. *maydis*. These findings suggest a systematic NHR against non-adapted pathogens in soybeans. This response was characterized by an overlap between metabolic processes and response to stimulus. Several metabolic processes provide the soybean with innate immunity to the non-adapted pathogen, *B*. *maydis*. This research investigation on NHR in soybeans may foster a better understanding of plant innate immunity, as well as the interactions between plant and non-adapted pathogens in intercropping systems.

## Introduction

Nonhost resistance (NHR) is a plant immune response against major microorganisms that are pathogenic to other plant species [1, 2]. Unlike *R*-gene-mediated resistance, which is governed by either a single or a few genes, NHR is mainly controlled by multiple genes expressed in multiple patterns and durable defense responses [2]. Due to its highly robust and exceptional resistance properties, NHR is of scientific and economic importance. Information regarding the mechanism underlying NHR may serve as a foundation for the development of novel strategies for crop disease management and crop distribution [3].

Soybeans are an important crop throughout the world because of their high plant protein and oil content [4]. Growing a combination of different crops and species in one field can boost yield and suppress crop diseases [5, 6]. Maize/soybean intercropping is a common combination used in some countries to address issues of limited arable land and increasing food demand [6]. In the maize/soybean intercropping system, maize is susceptible to infection by *Bipolaris maydis*, whereas soybeans present no detectable symptoms of disease [7]. *B*. *maydis* does not have a soybean pathogen phenotype, nor is it related to any known soybean pathogens. In addition, it does not infect soybeans via touch interactions. A previous microscopy analysis of infected soybeans showed that successful callose deposition and hydrogen peroxide (H_2_O_2_) production (S1 Fig) may be the first line of defense against *B*. *maydis* growth [7]. Based on these findings, we hypothesized that soybean resistance to *B*. *maydis* is established via NHR, which is the most robust and durable forms of plant resistance in nature. It allows soybean plants to protect themselves against a wide variety of parasitic microorganisms [8–9].

In the past few decades, several studies have confirmed the existence of NHR in plants [7, 9–12]. Unlike the well-studied host resistance conferred by plant resistance (*R*) genes, the molecular basis of NHR remains elusive. In this present study, the foundation of soybean NHR against *B*. *maydis*, a pathogen affecting a remotely related plant species, was characterized at the proteomic level. The availability of complete soybean genome sequences and recent developments in various sequencing technologies have allowed us to investigate the complex mechanisms underlying NHR in soybeans at the protein level [13–15].

Plants respond to stresses via differential expression of sets of genes, which results in changes in protein levels. The proteome represents the global protein expression profile of a species at a given time and under a set of conditions [16, 17]. Proteomic analysis involves identifying proteins involved in stress responses, determining their functions, and identifying possible regulatory networks that can be used to interpret stress-responsive processes that occur in plants [16, 17].

In a previous study, although there were no visible symptoms in soybean leaves inoculated with *B*. *maydis*, microscopic observations showed that successful callose deposition and H_2_O_2_ production (S1 Fig) may be the immediate response of a plant to restrict the growth of *B*. *maydis* [7]. Information on the molecular mechanism underlying this kind of robust and durable defense in whole soybean plants is currently limited. To better understand the mechanism of NHR in soybean, it is important to identify proteins that might be involved in the soybean response to exposure to *B*. *maydis*, determine the types of metabolic proteins involved in the NHR in soybeans, compare the findings of the present study and those of previous NHR investigations in plants, and determine whether any organelle was involved in the response to *B*. *maydis*. This present study addresses these issues and summarizes the metabolic processes involved in NHR in soybeans. The results may facilitate further our understanding of NHR in soybean plants.

## Materials and Methods

### Plant growth and inoculation

Seeds of the soybean cultivar Hua Chun 6 were sown in floriculture substrate in pots [15 cm (D) × 15 cm (H)] and grown in a greenhouse (70% relative humidity, 25°C/20°C day/night temperature, 14 h/10 h photoperiod) at a density of two plants per pot. These pots contained a mixture of sand and floriculture substrate at a ratio of 1:1 [18]. Samples of *B*. *maydis* race O (Y664) were collected from Wenshan Prefecture (east longitude 104°35′ and northern latitude 23°18′) in China’s Yunnan Province and preserved at the Yunnan Agricultural University College of Plant Protection. The *B*. *maydis* isolate was grown for 8 days at 27°C in a culture medium containing potato dextrose agar (PDA) and in the dark. The PDA medium was prepared as described elsewhere [19]. Conidia were collected with sterilized water (with 0.1% Tween-20) and the concentration was adjusted to 1 × 10^5^ conidia per mL. This mixture served as the inoculating solution. Then, 200 mL of sterilized water (with 0.1% Tween-20) was used for mock inoculation of the controls, and 200 mL of the conidia solution was used to inoculate the treatment groups. At 72 h post-inoculation, soybean leaves, stems, and roots were collected from both *B*. *maydis*-inoculated and mock-inoculated (control) soybean seedlings, and the stems and roots were cut into 1-cm sections for easier grounding. Three replicates were prepared for each experiment.

### Protein extraction from soybean leaves

One gram of leaf tissue (1.5 g of stem and root tissue) was ground to a fine powder using a mortar and pestle and in liquid nitrogen. The powder was transferred to a 50-mL centrifuge tube and supplemented with 20 mL of a pre-cooled mixture of 10% trichloroacetic acid and 2 mM phenylmethanesulfonyl fluoride (PMSF) and 50 mM DL-dithiothreitol (DTT), vortex mixed with acetone, and incubated overnight (or for 16 h–18 h) at -20°C. After incubation, the suspension was centrifuged at 20,000 rpm for 20 min at 4°C, and the protein pellet was washed three times with 2 mM PMSF and 50 mM DTT in 80% acetone. The pellet was dried with a circulating water vacuum pump (SHZ-IIID, Shanghai, China). A 20-mg fraction of the protein powder was then resuspended in 450 μL of lysis buffer (7 M urea, 2 M thiourea, 5% (3-[(3-cholamidopropyl)dimethylammonio]propanesulfonate (CHAPS), 2 mM DTT, and 0.3% Biolyte) by flicking the tube wall with fingers, and then incubated for 1 h at 25°C (or room temperature). The suspension was later centrifuged at 22,000 rpm for 20 min at 25°C, and then the resulting supernatant was collected. The protein content of the samples was quantitated using the Bradford method with bovine serum albumin as protein standard [20]. For each treatment, three independent proteins were prepared, and triplicate 2-DE gels were used for each sample.

### 2-DE used for protein separation

Six samples were individually run in triplicate to control gel variation. Approximately 600 μg of the purified protein extract was adjusted to a total volume of 500 μL with a rehydration buffer containing 7 M urea, 5% (w/v) CHAPS, 0.5% (v/v) immobilized pH gradient (IPG) buffer (pH 4–7 NL) (GE Healthcare, Munich, Germany), and freshly prepared 20 mM DTT for every 24-cm IPG strip (GE Healthcare, Munich, Germany). The adjusted protein solution was vortexed and centrifuged for 5 min at 22,000 rpm at 4°C and then was directly loaded into a focusing tray. IPG strips (4–7 NL, 24 cm; GE Healthcare, Munich, Germany) were passively rehydrated for 14 h at 20°C. Then, the proteins (three replicates per treatment) were separated in the first dimension by isoelectric focusing (IEF), and then in the second dimension via sodium dodecyl sulfate-polyacrylamide gel electrophoresis (SDS-PAGE).

IEF was conducted using the IPGphor system (GE Healthcare, Munich, Germany) under the following conditions [21–22]: 250 V for 30 min with a linear ramp, 1,000 V for a minimum of 1 h with a rapid ramp, 9,000 V for 4.5 h with a linear ramp, and finally, 9,000 V at 65,000 V/h with a rapid ramp; the entire system was kept at 20°C. After IEF, the strips were equilibrated with 6 M urea, 2% SDS, 1.5 M Tris-HCl (pH 8.8), 30% glycerol, 0.02% bromophenol blue, and 130 mM DTT for 20 min. The second equilibration step was performed in a solution consisting of 6 M urea, 2% SDS, 1.5 M Tris–HCl (pH 8.8), 30% glycerol, 0.02% bromophenol blue, and 135 mM iodoacetamide (IAA) for 20 min. The equilibrated strips were placed on 12.5% SDS-polyacrylamide gels and sealed with 1% low-melting agarose. SDS-PAGE was performed at 20 mA/gel first and then adjusted to a constant current of 30 mA/gel after 45 min. The 2-DE gels were fixed with the mixture solution (40% ethanol, 10% acetic acid) for 3 h at 16°C, and then stained overnight with Coomassie brilliant blue (CBB) G-250. The protein spots were detected by destaining with a mixture solution (5% w/v ammonium sulfate and 0.5% methanol).

### SDS-PAGE analysis

Gels were then carefully washed with double-distilled water, scanned at an optical resolution of 300 dpi (or 600 dpi), and saved as.tiff files. For spot detection and volume quantification, 300 dpi.tiff files were analyzed using the PDQuest software version 7.4. To correct for variability in CBB staining, the individual spot volumes were normalized by dividing each spot’s optical density (OD) value by the sum total OD of all the spots in the respective gels. Automated and manual spot matching were also performed [23].

The relative volumes were used to identify individual spots that were significantly differentially expressed between the two groups of gels (at least two-fold higher or lower and statistically significant as calculated by the student’s *t*-test, at *P* < 0.05) (|ratio|≥2, *P* < 0.05). Only those with significant and reproducible changes were considered differentially expressed proteins [21].

### Protein spot in-gel digestion, identification, and prediction of protein function

Soybean leaf, stem, and root proteins with differential expression patterns (|ratio|≥2, *P* < 0.05) on gels were manually excised, washed three times with Millipore^®^ pure water, and subjected to in-gel digestion and MALDI-TOF/TOF MS analysis, as described elsewhere [21,22]. The resulting peptide sequence data were submitted to the online MASCOT search engine (Matrix Science, London, U.K.) (http://www.phytozome.net/soybean) and queried against NCBInr databases [22].

The minimum requirements of our protein analysis were as follows: (i) at least three peptide sequences with identities higher than the threshold matched; and (ii)≧9% coverage of the total protein sequence by matching peptide-positive matches were BLASTP searched against the NCBI protein database (http://www.ncbi.nlm.nih.gov) for updated annotation and identification of homologous proteins [22, 23]. Some of the predicted cellular locations were determined using the Plant-PLoc version 2.0 (http://www.csbio.sjtu.edu.cn/bioinf/plant-multi/). STRING (http://www.string-db.org/) was used for predicting protein-protein interactions

## Results

2-DE was used to screen for differentially expressed proteins that were associated with *B*. *maydis* infection. A total of 68 proteins were differentially expressed in the roots, stems, and leaves of soybeans at 72 h after inoculation. Some of these 68 proteins were similar to those of NHR components reported in previous studies, whereas others differed from those earlier described [9].

### Sixteen proteins in soybean leaves were induce after exposure to *B*. *maydis*

**Fig 1:**
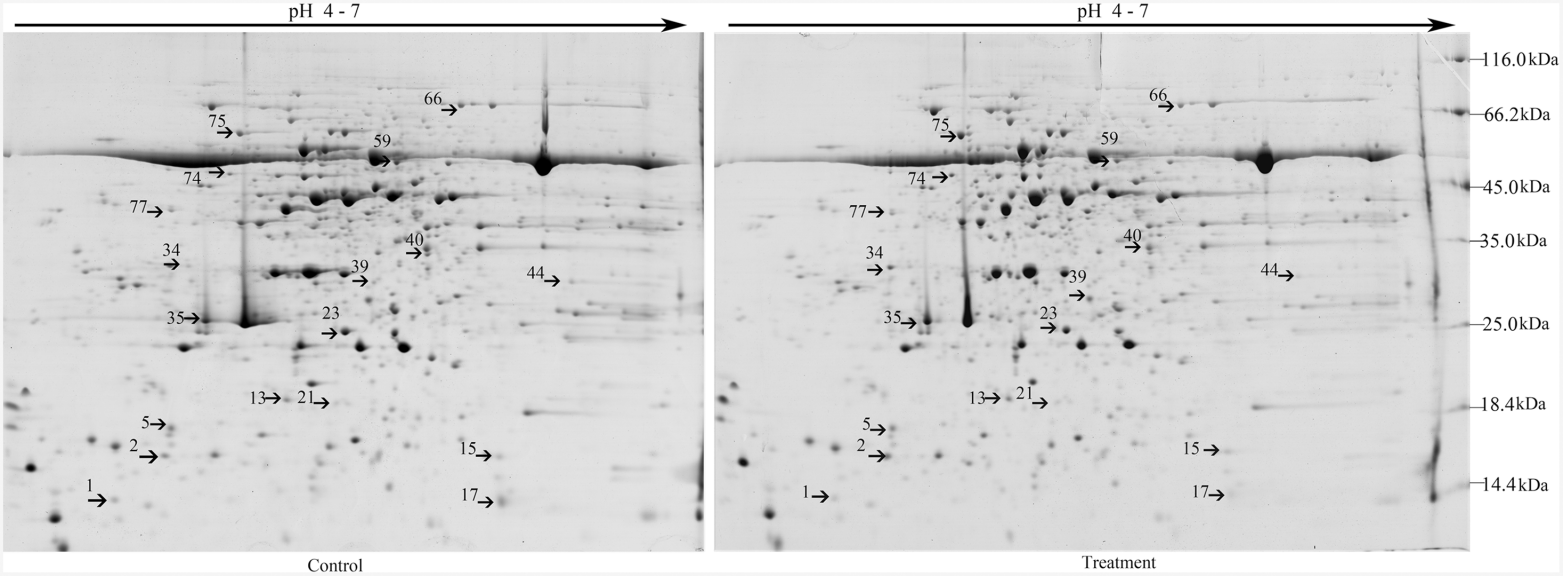
Representative 2-DE gel pattern of soybean leaves at 72 h post-inoculation with *B*. *maydis*. For isoelectric focusing, a total of 500 μg/600 μL proteins were loaded onto each 24-cm IPG strip (pH 4–7). This was followed by SDS-PAGE on a 12.5% gel and Coomassie staining. 2-DE maps of proteins from untreated control leaves or leaves treated with *B*. *maydis* are shown. Proteins differentially regulated in response to *B*. *maydis* were numbered in pairs of control and treatment maps. The MW (kDa) and pI of each protein was determined using a 2-DE marker.

Leaf proteomic analysis of soybean NHR was performed to determine the mechanism underlying the response of soybean plants to *B*. *maydis*. Approximately 1,300 ± 50 protein spots were separated on 2-DE gels (Fig 1).

The 18 protein spots showed significant changes (|ratio| > 2, *P* < 0.05). Among these, 16 proteins were further classified by using MALDI-TOF-MS as provided by AmiGO into five groups, including proteins related to metabolic processes, catalytic activity, developmental processes, cellular components (such as structural proteins), and defense response (Fig 1, Table 1). Eight of the nine metabolic proteins were located in chloroplasts. Of these, 6 proteins showed increased staining intensity and 2 proteins showed decreased staining intensity.

**Table 1:** Predicted functions and subcellular locations of soybean leaf proteins in response to *B*. *maydis*.

Spot No.	(a) Up (+) /down (-)	Ratio (b)	Protein score	SC (%) (c)	PM (d)	Mr(e) theoretical/ observed	P*I (*f) theoretical/ observed	Function (g)	Species (h)	GenBank Acc. No. (i)	Predicted cellular location (j)
**Metabolic process**											
23	+	2.8	180	32.93	6	26.77/31.1	6.21/5.14	Chlorophyll a-b binding protein 6A Chloroplastic-like	*Glycine max*	gi|356559472	Chloroplast
2	+	13.3	105	35.97	3	11.44/14.36	4.36/4.52	Unknown	*Glycine max*	gi|255626375	Chloroplast
15	+	4.4	487	76.97	12	20.23/23.2	8.87/6	Uncharacterized protein	*Glycine max*	gi|351725817	Chloroplast
39	+	2.0	289	38.87	9	32.5/36.84	5.93/5.32	Unknown	*Glycine max*	gi|255641907	Chloroplast
59	+	2.1	307	61.97	12	30.24/56.14	5.43/5.92	Unnamed protein product	*Glycine max*	gi|257670630	Chloroplast Mitochondrion
66	-	5.7	189	35.94	11	49.87/56.14	5.4/5.92	BAHD acyltransferase DCR-like	*Glycine max*	gi|356530840	Cytoplasm
77	-	5.6	178	43.94	16	52.39/66.01	5.26/5.38	4-hydroxy-3-methylbut-2-enyl diphosphate reductase-like	*Glycine max*	gi|356538819	Chloroplast
5	-	2.6	118	21.8	4	26.33/18.19	6.93/4.65	Ribulose-1,5-bisphosphate Carboxylase/oxygenase large subunit	*Glycine max*	gi|290586534	Chloroplast
44	+	4.1	583	56.42	23	53.03/50.09	6/5.58	Ribulose-1,5-bisphosphate carboxylase/oxygenase large subunit	*Glycine max*	gi|91214125	Chloroplast
**Developmental process**											
34	+	2.5	422	55.26	10	35.27/23.52	6.66/5.37	Oxygen-evolving enhancer protein 1 Chloroplastic-like	*Glycine max*	gi|356559442	Chloroplast
**Response to stimulus**											
13	+	7.7	1020	148.8	13	17.47/20.58	5.41/5.9	Unknown	*Glycine max*	gi|255626437	Cytoplasm
35	+	2.3	92	21.15	5	32.19/34.2	5.01/5.3	Acidic chitinase	*Glycine max*	gi|4835584	Vacuole
**Cellular component organization or biogenesis**											
74	-	2.7	741	91.39	26	54.27/68.09	4.92/6.51	V-type proton ATPase subunit B 1-like isoform 2	*Glycine max*	gi|356536394	Cytoplasm
75	+	2.6	352	62.97	16	54.29/70.09	4.95/5.13	V-type proton ATPase subunit B 2-like isoform 2	*Glycine max*	gi|356568551	Cytoplasm
**Defense response**											
21	+	2.1	179	64.29	6	18.25/22.11	6.12/5.08	Trypsin inhibitor	*Glycine max*	gi|9367042	Vacuole
**Catalytic activity (molecular function)**											
17	+	9.2	320	52.35	6	16.40/15.69	6.91/5.19	Nucleoside diphosphate kinase	*Glycine max*	gi|26245395	Mitochondrion Nucleus

(a) Protein spots increased (+) or decreased (-) in staining intensity in response to *B*. *maydis*

(b) Ratio was equal to the relative expression of (Treatment / Control)

(c) SC, sequence coverage by PMF

(d) PM, number of peptides matched

(e) Theoretical molecular mass was from report of identification and NCBI database, and experimental molecular mass was observed from real 2-DE gel image combined with analysis of PDQuest software

(f) Theoretical isoelectric point was from reports and from the NCBI database; experimental isoelectric points were observed from real 2-DE gel images combined with analysis of PDQuest software

(g) Predicted function of the protein obtained via the MASCOT software from the NCBI database

(h) Species of the proteins obtained via the MASCOT software from the NCBI database

(i) GenBank Acc. No., accession number in NCBI database

(j) Plant-mPLoc: Predicting subcellular localization of plant proteins including those with multiple sites (predicted at the website: http://www.csbio.sjtu.edu.cn/bioinf/plant-multi/)

Nucleoside diphosphate kinase (here considered indicative of catalytic activity) was located in the nucleus, and its concentration increased by 9.2-fold. The spot representing the stress-response protein oxygen-evolving enhancer protein 1 increased in staining intensity [24]. The spots representing 2 defense proteins, including acidic chitinase and trypsin inhibitor, also increased in staining intensity, mainly in the vacuole. The intensity of spot 74, which represented the V-type proton ATPase subunit B 1-like isoform 2, showed a decrease in staining intensity in the cytoplasm, whereas that of its subunit, B 2-like isoform 2, increased (Table 1). Some of these differentially expressed proteins are involved in metabolic processes, developmental processes, organization of cellular components, and catalytic activity. Expression of these particular proteins in soybean leaves was induced in response to *B*. *maydis*, together with the production of acidic chitinase and trypsin inhibitor.

### Twenty-five proteins were expressed in the stems of soybean plants in response to *B*. *maydis*

**Fig 2:**
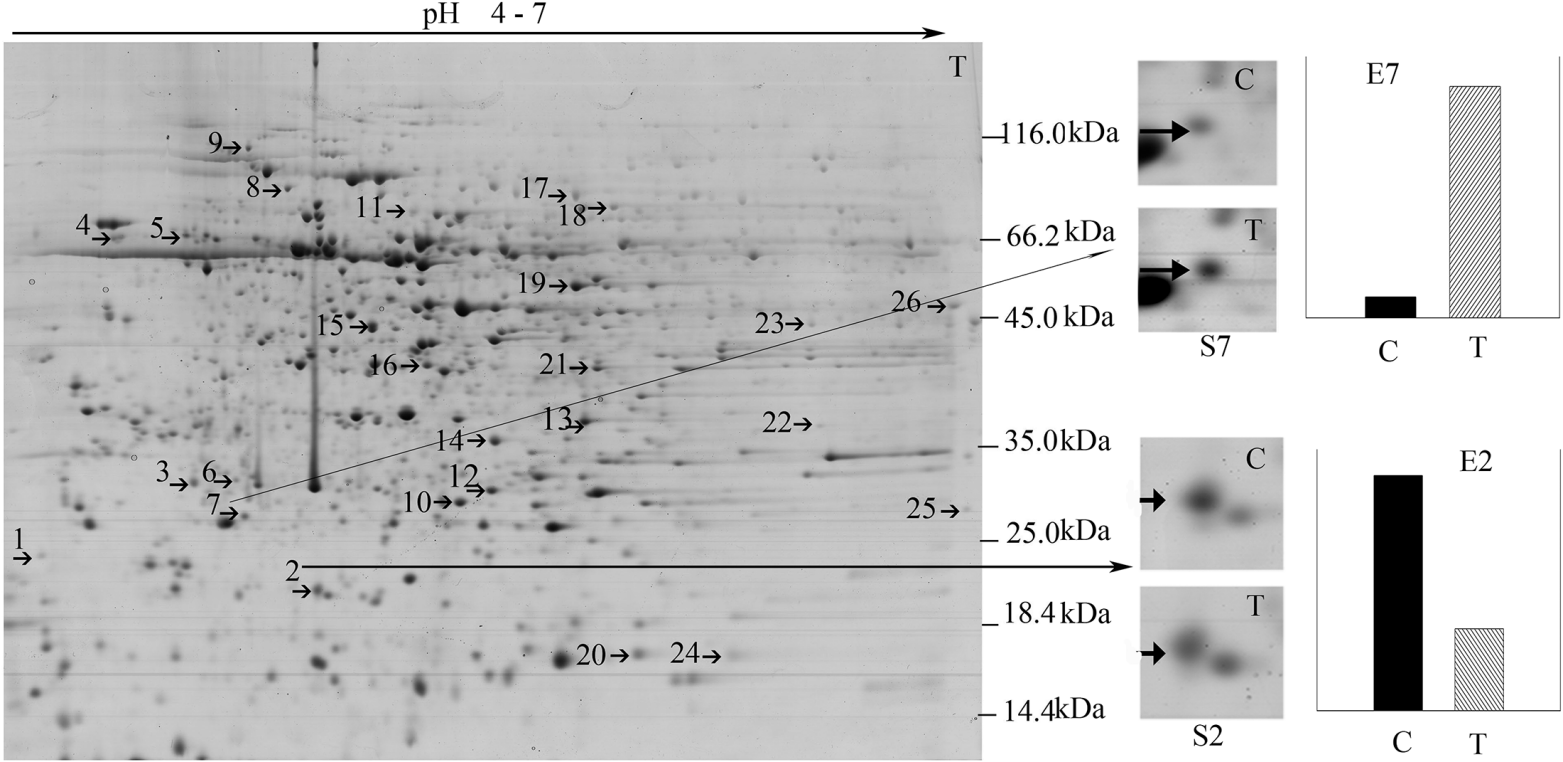
Two DE patterns of stem proteins responding to *B*. *maydis*. S7 and S2 show enlarged images of protein spots 7 and 2, which were differentially expressed under *B*. *maydis* stress conditions; “C” refers to the protein pattern dissolved on the control gel, and “T” to the protein pattern dissolved on the treatment gel. The differential abundance of proteins was read using the PDQuest software and is plotted as the relative intensity enumerated in E7 and E2. E7 and E2 represent the relative expression of protein spots 7 and 2 in the control and treatment groups. “C” also refers to the control, and “T” to treatment. S7/E7 and S2/E2 are representative images of the differentially expressed proteins in the stems of soybean plants.

Several studies have documented that leaf and root proteomics can be used to interpret the responses of soybeans to biotic and abiotic stress [24]. However, the stem protein response to pathogens is remains elusive. Here, 72 hpi stem proteomic analysis was performed to produce a more systemic understanding of how the three major organs (roots, stems, and leaves) of soybean plants are involved in the responses to the non-adapted pathogen *B*. *maydis*. Approximately 1,000 ± 50 protein spots were separated on 2-DE gels (Fig 2).

Among these 26, 25 differentially expressed (|ratio| > 2, *P* < 0.05) proteins were successfully identified and sorted into five functional groups, including proteins associated with metabolic processes, cellular components, defense response, developmental processes, and molecular function (Table 2). Here, 48% of the stem proteins were determined to be involved in metabolic processes. The spots associated with half of these showed an increase in staining intensity with *B*. *maydis* infection, whereas half showed a decrease. Around 16% of the evaluated stem mitochondrial proteins showed a change in expression after infection, suggesting that NHR in soybeans is involved in significant energy consumption. Two of the mitochondrial proteins were related to heat shock 70, which is the protein chaperone that is responsible for the refolding and misfolding of proteins. A putative cysteine protease was grouped into the metabolic process group, although a previous study reported that it is involved in both developmental and pathogen-related PCD [25]. Protein spots 4, 8, and 19 had functional annotations similar to those of Hsp70, although these were classified in different functional groups and were predicted to localize to different locations (mitochondrion and chloroplast), which was consistent with the diverse locations and functions of Hsps. The staining intensity of spot 19 (Hsp 70) showed a 12-fold increase relative to that of the controls.

**Table 2:** Predicted functions and subcellular locations of soybean stem proteins responsive to *B*. *maydis*.

Number of spots	(a) Up (+) /down (-)	Ratio (b)	Protein score	SC (%) (c)	PM (d)	Mr(e) theoretical/ observed	P*I (*f) theoretical/ observed	Function (g)	Species (h)	Acc. no. (i)	Predicted cellular location (j)
**Metabolic process**											
1	-	2	116	35.43	4	14.27/25.56	4.13/4.11	C2 domain-containing protein At1g63220-like	*Glycine max*	gi|356522642	Cytoplasm
3	-	3	99	20.28	5	39.70/31.44	7.01/4.62	Putative cysteine protease	*Glycine max*	gi|149393486	Vacuole
8	-	2	182	27.88	14	73.38/72.28	5.37/4.73	Heat shock cognate 70 kDa protein 2-like	*Glycine max*	gi|356502432	Mitochondrion
9	-	6	551	41.03	27	93.29/84.63	4.86/4.9	Endoplasmin homolog	*Glycine max*	gi|356564371	Endoplasmic reticulum
10	+	3	336	64.75	14	33.79/31.41	5.84/5.18	Isopentenyl-diphosphate Delta-isomerase	*Glycine max*	gi|356531894	ChloroplastNucleus
16	+	2	543	47.52	18	54.53/51.75	5.89/5.54	Mitochondrial-processing peptidase subunit alpha-like	*Glycine max*	gi|356522822	Mitochondrion
18	+	2	1030	95.05	25	47.91/48.5	5.49/5.76	Enolase-like	*Glycine max*	gi|356505318	Cytoplasm
19	+	12	484	42.84	24	72.68/69.06	5.82/5.53	Heat shock 70 kDa protein	*Glycine max*	gi|356524786	Mitochondrion
21	+	3	84	11.08	3	38.34/40.41	5.3/5.83	Adenosine kinase 2-like	*Glycine max*	gi|356505238	Mitochondrion
22	-	2	192	25.26	5	33.13/33.89	6.14/6.52	Uncharacterized protein LOC100781644	*Glycine max*	gi|356563399	Cell wallCytoplasm
25	-	3	436	18.45	7	22.92/23.69	9.1/6.89	Peptidyl-prolyl cis-trans isomeraseCYP20-2, chloroplastic-like isoform 2	*Glycine max*	gi|356576933	ChloroplastEndoplasmic reticulum
13	+	2	233	62.95	12	30.81/31.24	5.9/5.58	Carbonic anhydrase, chloroplastic-like	*Glycine max*	gi|356501896	Chloroplast
**Cellular component**											
2	-	3	98	25.7	4	23.77/24.03	5.55/4.62	Uncharacterized protein LOC100305490	*Glycine max*	gi|351727673	Chloroplast
11	-	3	112	20.00	7	55.78/57.38	5.15/5.14	ATP synthase CF1 alpha subunit	*Glycine max*	gi|91214148	Chloroplast
14	+	10	259	59.75	14	35.30/40.1	5.47/5.54	Probable carboxylesterase 2-like	*Glycine max*	gi|356521488	Nucleus
17	+	2	366	46.25	15	46.56/51.98	5.61/5.7	26S protease regulatory subunit 6B homolog	*Glycine max*	gi|356539717	CytoplasmNucleus
24	-	6	396	51.01	6	16.49/14.48	5.93/6.85	Nucleoside diphosphate kinase	*Glycine max*	gi|351720837	Nucleus
**Defense response**											
4	-	4	346	37.56	17	71.20/72.59	5.1/4.59	Heat shock cognate 70 kDa protein-like	*Glycine max*	gi|356569000	Chloroplast
6	-	2	743	56.86	11	29.00/29.76	6.34/4.8	Unknown	*Glycine max*	gi|255644428	Chloroplast
12	+	4	472	96.95	10	17.20/23.8	5.61/5.66	Unknown	*Glycine max*	gi|255628729	Chloroplast
20	+	3	847	141.35	13	17.50/17.73	5.64/5.97	Unnamed protein product	*Glycine max*	gi|227247706	Cytoplasm
23	-	3	522	55.43	11	36.88/39.24	8.18/6.49	Malate dehydrogenase 1 mitochondrial-like	*Glycine max*	gi|356543225	Mitochondrion
26	-	2	459	36.52	10	36.20/39.53	8.22/6.86	Malate dehydrogenase mitochondrial-like	*Glycine max*	gi|356517066	Mitochondrion
**Developmental process**											
7	+	11	834	79.46	16	35.27/28.73	6.66/4.87	Oxygen-evolving enhancer protein 1 chloroplastic-like	*Glycine max*	gi|356559442	Chloroplast
**Molecular_function**											
15	+	3	476	60.87	18	42.25/42.22	5.52/5.66	Alpha-1,4-glucan-protein synthase	*Glycine max*	gi|356495127	Golgi apparatus

(a) Protein spots increased (+) or decreased (-) in staining intensity in response to *B*. *maydis*

(b) Ratio was equal to the relative expression of (Treatment / Control)

(c) SC, sequence coverage by PMF

(d) PM, number of peptides matched

(e) Theoretical molecular mass was from report of identification and NCBI database, and experimental molecular mass was observed from real 2-DE gel image combined with analysis of PDQuest software

(f) Theoretical isoelectric point was from reports and from the NCBI database; experimental isoelectric points were observed from real 2-DE gel images combined with analysis of PDQuest software

(g) Predicted function of the protein obtained via the MASCOT software from the NCBI database

(h) Species of the proteins obtained via the MASCOT software from the NCBI database

(i) GenBank Acc. No., accession number in NCBI database

(j) Plant-mPLoc: Predicting subcellular localization of plant proteins including those with multiple sites (predicted at the website: http://www.csbio.sjtu.edu.cn/bioinf/plant-multi/)

This phenomenon indicates that NHR in soybean is related to protein functional diversity. Protein spot 10 (isopentenyl-diphosphate delta-isomerase), protein spot 17 (26S protease regulatory subunit 6B homolog), and protein spot 25 (peptidyl-prolyl *cis-trans* isomerase CYP20-2, chloroplastic-like isoform 2) were predicted to have more than one subcellular location. Protein spot 7 (oxygen-evolving enhancer protein 1, chloroplastic-like) was found to have been caused by the same protein as leaf protein spot 34, which showed a 2.5-fold increase in expression in leaves and a 11-fold increase expression in stems. This finding suggests that protein spot 34 might play an important role in soybean response to *B*. *maydis*. The staining intensity of protein spot 14 increased by 10-fold and that of protein spot 24 decreased by 6-fold in the nuclei of soybean stems. All 25 stem proteins were predicted in various important subcellular locations, including the nucleus, cytoplasm, endoplasmic reticulum, Golgi apparatus, vacuole, chloroplast, and mitochondrion, which indicated that the response proteins might include proteins related to metabolism.

### Root proteins activate metabolic processes in response to *B*. *maydis* challenge on leaves

**Fig 3:**
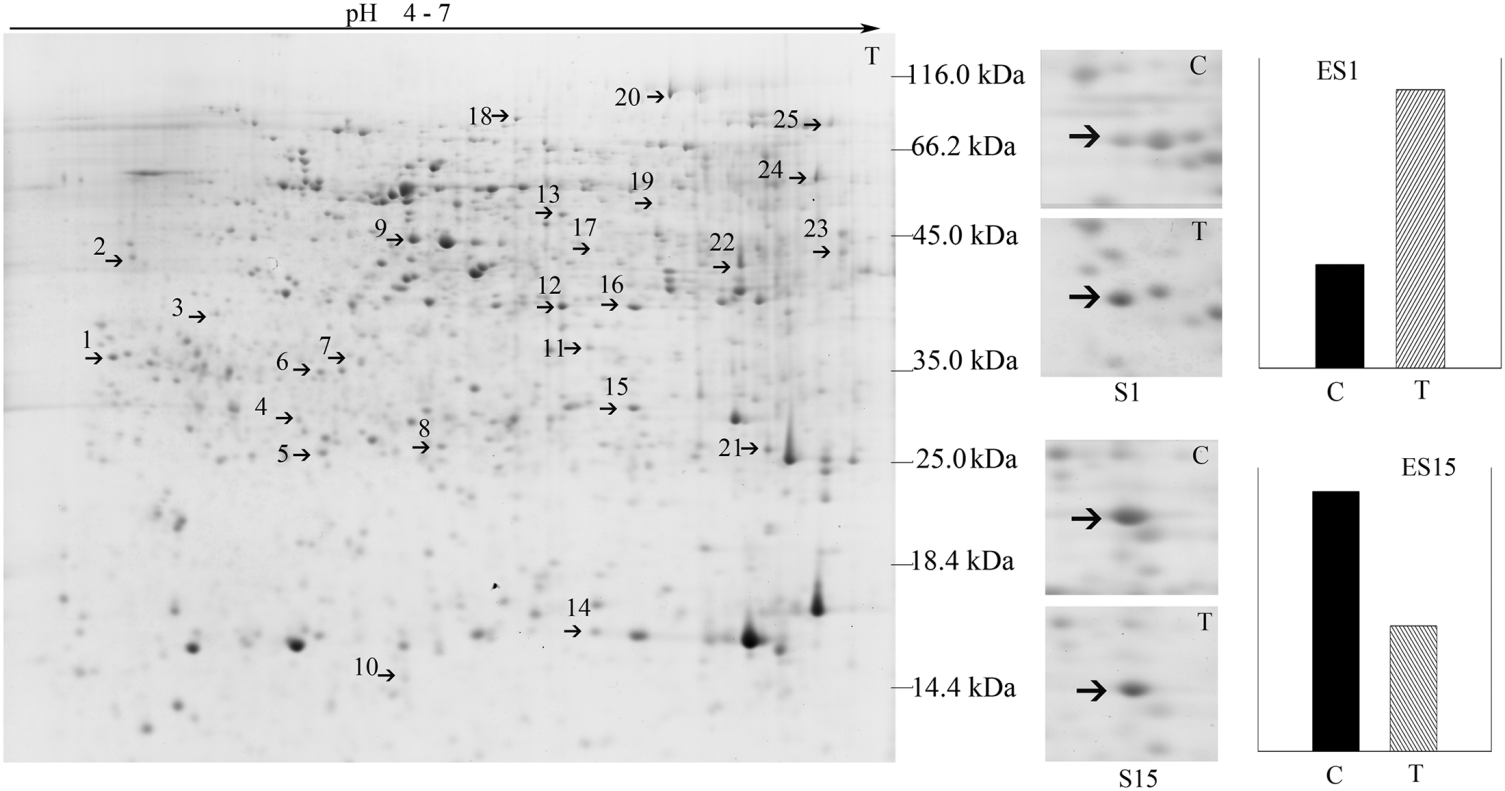
Two-DE map of root proteins responding to *B*. *maydis*. S1 and S15 were zoomed in the protein spot 1 and spot 15 differentially expressed under the *B*. *maydis* stress; “C” refers to the protein spot excised from the control gel, and “T” refers to the protein spot excised from the treatment gel. The differential abundance of proteins was determined using the PDQuest software and was plotted as the relative intensity enumerated as ES1 and ES15. ES1 and ES15 represent the relative expression between control and treatment of protein spot 1 and spot 15, “C” means control, and “T” indicates treatment. S1/ES1 and S15/ES15 are representative differentially expressed root proteins.

We investigated the patterns of root protein expression in response to *B*. *maydis* challenge on leaves (Fig 3).

Almost 1,000 proteins were detected in the CSS gel using PDQuest software 7.4, and all replicate gels showed highly similar distribution patterns in the 2-DE images. Around 25 proteins were differentially expressed (|ratio|≥2, *P* < 0.05). Overall analysis revealed that out of all the protein spots that matched in the treatment and control groups, 39% showed increased intensity and 61% presented a decrease in intensity. Twenty-three of these differentially expressed proteins were successfully identified in six functional groups, including proteins related to metabolic processes, cellular components, regulation of cellular processes, transmembrane transport, defense response, and molecular function (Table 3). These proteins were detected in the nucleus, cytoplasm, cell wall, cell membrane, mitochondrion, and Golgi apparatus of root cells in response to a *B*. *maydis* challenge on leaves, which might contribute to the multiple NHR responses to non-adapted pathogens.

**Table 3:** Proteins in soybean roots in response to the challenge of *B*. *maydis* on leaves.

No. of spots	Up (+)/down (-)	Ratio	Protein score	SC (%)	PM	Species	Acc. no.	Protein name	Predicted cellular location
**Metabolic process**									
1	+	2.7	923	69.6	12	*Glycine max*	gi|356516563	Elongation factor 1-delta-like	Mitochondrion
4	+	2.2	262	32.3	7	*Glycine max*	gi|255646685	Unknown	Chloroplast
5	+	2.4	502	57.9	8	*Glycine max*	gi|351723871	Chalcone isomerase 4-like	Chloroplast
8	+	2.1	639	37.8	8	*Glycine max*	gi|356507848	Proteasome subunit beta type-6-like	Nucleus
17	-	2.5	322	34.3	9	*Glycine max*	gi|356516166	Caffeic acid 3-O-methyltransferase-like isoform 1	Chloroplast
19	+	16.7	527	47.6	15	*Glycine max*	gi|356543373	elongation factor 1-gamma-like	Cytoplasm
22	+	2.4	721	50.0	16	*Glycine max*	gi|356509324	Alcohol dehydrogenase 1-like	Cytoplasm
24	-	2.4	519	68.2	14	*Glycine max*	gi|356505332	Fructose-bisphosphate Aldolase,cytoplasmic isozyme	Cytoplasm
25	+	33.1	500	21.3	9	*Glycine max*	gi|351721496	Copper amino oxidase precursor	Cell wall
**Cellular component**									
9	-	2.1	694	67.9	16	*Glycine max*	gi|1498340	Actin	Cytoplasm
10	-	3.7	432	45.1	12	*Glycine max*	gi|255641658	Unknown	Cytoplasm
11	-	3.6	364	42.3	11	*Glycine max*	gi|356509393	L-ascorbate peroxidase T, chloroplastic-like isoform 1	Chloroplast Mitochondrion
18	-	2.9	666	39.7	15	*Glycine max*	gi|356532109	2,3-bisphosphoglycerate-independent phosphoglycerate mutase-like	Cytoplasm
**Regulation of cellular process**									
2	-	6	112	57.4	3	*Glycine max*	gi|356560765	Cnkyrin repeat domain-containing protein 2-like	Chloroplast
3	-	2.1	276	30.8	7	*Glycine max*	gi|356566211	Proliferating cell nuclear antigen-like	Nucleus
21	-	2.1	498	64.6	11	*Glycine max*	gi|356537146	Ubiquitin-conjugating enzyme E2 variant 1D-like	Nucleus
**Transport**									
6	+	2.2	207	38.0	7	*Glycine max*	gi|356556130	Plasma membrane-associated cation-binding protein 1-like	Cell membrane
**Response to stimulus (defense response)**									
12	-	2.9	219	9.29	2	*Glycine max*	gi|359807323	Uncharacterized protein LOC100814078	Chloroplast. Mitochondrion
14	-	3.8	700	75.3	9	*Glycine max*	gi|351724985	Uncharacterized protein LOC100499771	Cytoplasm
16	-	3	458	36.7	11	*Glycine max*	gi|356538212	Isoflavone reductase-like	Cytoplasm
20	+	3.5	930	44.3	30	*Glycine max*	gi|351726848	Seed linoleate 9S-lipoxygenase	Cytoplasm
**Catalytic activity (molecular function)**									
13	-	3.4	366	48.9	14	*Glycine max*	gi|356495127	Alpha-1,4-glucan-protein synthase [UDP-forming]-like	Golgi apparatus
23	-	2.8	323	29.2	8	*Glycine max*	gi|356505967	Auxin-induced protein PCNT115-like isoform 2	Chloroplast

Protein spots 1 and 19 were identified as elongation factor 1-delta-like (2.7-fold increase) and elongation factor 1-gamma-like (16.7-fold increase) proteins, which were predicted to localize to the mitochondrion and cytoplasm, respectively. This indicated that the metabolic processes related to protein synthesis in soybean roots might be different in response to *B*. *maydis*.

Protein spot 25, which is a copper amino oxidase precursor, showed a significant increase in intensity (33.1-fold) in soybean root organs. Chalcone isomerase 4-like, proteasome subunit beta type-6-like, and alcohol dehydrogenase 1-like proteins are related to flavone and alcohol metabolic processes, showed an increase in spot intensity in response to *B*. *maydis*, which indicated that several proteins in the roots might be bifunctional under stress conditions.

The spot intensities of proliferating cell nuclear antigen-like and auxin-induced protein decreased, suggesting that the soybean root cells underwent a reduction in growth to increase its defense response to the infection. On the other hand, the intensity of protein spot 8, which is related to a protease, increased. This finding indicated that the protein degradation process also increased in response to *B*. *maydis* (Table 3).

In summary, almost all of the plant cell organelles were predicted to respond to the *B*. *maydis* challenge on leaves, and the biological processes involved in growth and development were possibly bifunctional.

## Discussion

Competitive and facilitative interactions among different plant species have played major roles in shaping natural communities. For decades, this principle has been employed to increase crop yield [26, 27]. Plants sense their environments using chemicals, touch, and light [26, 27]. In maize/soybean intercropping systems, the transfer of *B*. *maydis* spores from maize to soybeans is a common occurrence. There have been several reports of interactions between maize and soybeans, and these have been extensively used to increase yield or improve soil fertility. However, there has been no report discussing the interactions between soybeans and *B*. *maydis*, which often come into contact in ecological networks involving maize and soybean intercropping systems. Therefore, the findings of the present study not only offer new insights into NHR in the soybean response to *B*. *maydis*, but also into the positive interactions mediated by *B*. *maydis* between soybeans and maize. This is the first detailed study on how *B*. *maydis* mediates interactions between maize and soybeans.

### Whole-plant resistance is invoked in almost all of cellular components of the soybean plant

**Fig 4:**
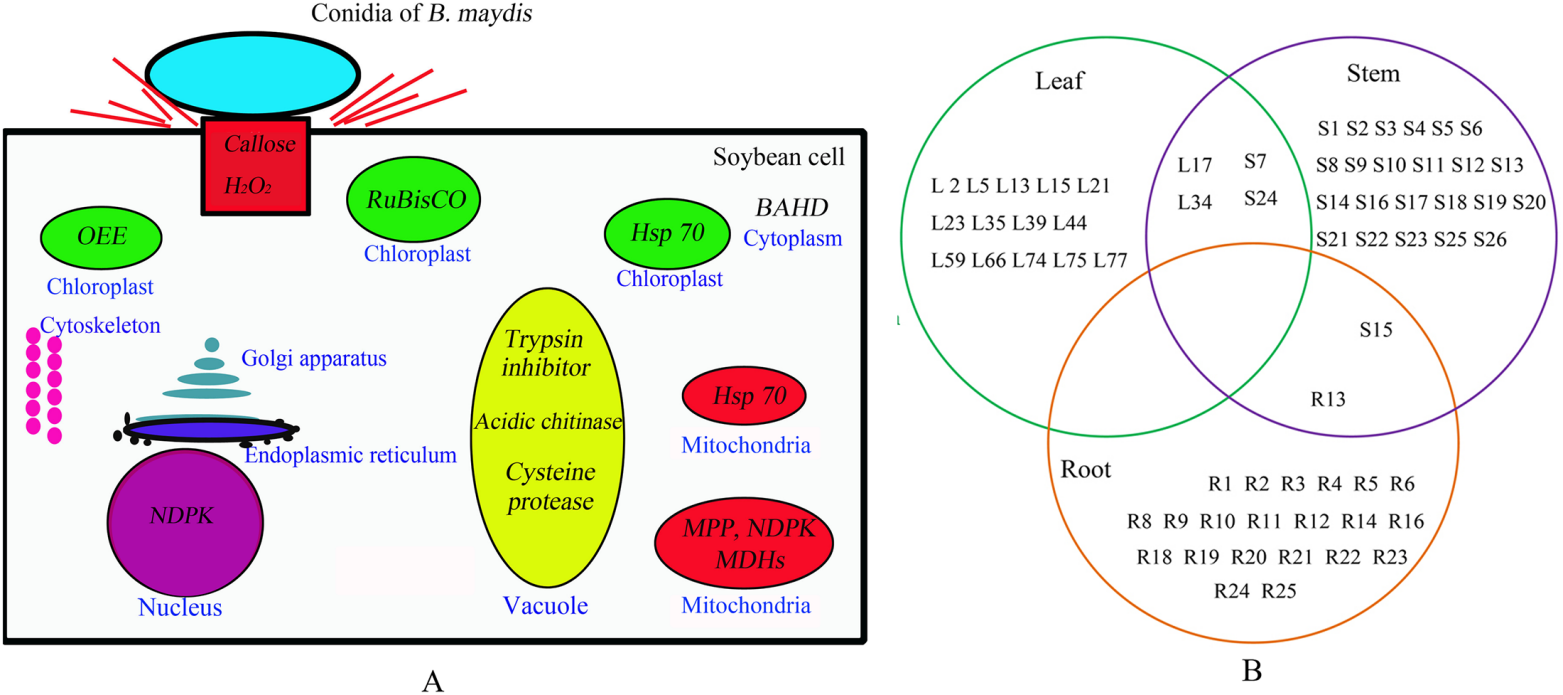
Proteins responsive to *B*. *maydis* and its predicted multiple subcellular locations. A) A conceptual diagram of a systematic NHR of soybean against *B*. *maydis*. H_2_O_2_ production and callose deposition were processed upon the interaction between soybean and *B*. *maydis* starting at the cell wall of soybean. The subcellular locations of the differentially expressed proteins were predicted. Changed proteins are shown in italics, and the organelle is written in blue. For instance, Hsp 70 was predicted to exist in chloroplasts and mitochondria. All of the major organelles responded to *B*. *maydis*. B) Venn diagram representing the differentially expressed proteins found in the leaves, stems, and roots of soybean plants. An L, S, or R character was placed before the spots (corresponding to the 2-DE pattern) corresponding to leaf, stem, and root proteins. Two pairs of leaf and stem protein spots were attributed to the same protein, L17 and S24, and L34 and S7. There was only one pair of stem and root protein spots that was attributed to the same protein, S15 and R13. However, no protein overlap between leaf and root was detected.

Although *B*. *maydis* was sprayed only onto the leaves of the soybean plants, proteins of the stem and roots also responded to this particular stimulus (Figs 1, 2, 3 and 4). These results suggest that the response of soybeans to *B*. *maydis* may not be limited to the site of exposure, and that the signal of the stimulus could be transmitted to remote organs. The level of expression of several proteins was reprogrammed far from the site of inoculation. Only one pair of root and stem protein spots consisted of the same protein, e.g., S (stem) 15 and R (root) 13, an alpha-1,4-glucan-protein synthase [UDP-forming] homolog. Two pairs of stem and leaf protein spots (L17 and S24) comprised the same protein, the NDK homolog. Leaf protein spot (L34) and stem protein spot (S7) contained an oxygen-evolving enhancer protein homolog. However, there was no protein overlap between leaves and roots. These results suggest that roots, stems, and leaves of soybeans could differentially respond to *B*. *maydis* challenge on leaves, which could render a whole plant resistant to *B*. *maydis*. Thus, almost all of the metabolic proteins were triggered in almost all subcellular structures. These included the cell wall, cell membrane, cell skeleton, cytoplasm, chloroplasts, mitochondria, nucleus, Golgi apparatus, and endoplasmic reticulum (Fig 4A). This is the first report to find evidence of a multiple-responsive NHR in soybeans against *B*. *maydis*, a pathogenic fungus of a phylogenetically distant crop.

### Proteins involved in NHR in soybean overlapped with metabolic proteins

NHR is strongly associated to basic plant metabolism, and host pathogens are known to target such primary metabolic pathways to establish virulence [2]. The results of the present study showed that proteins related to metabolic processes could be co-induced in response to non-adapted pathogens. For example, the ability of soybean plants to cope with *B*. *maydis* stress depends on a number of changes in their proteins, which the present study has determined to be reprogrammed after inoculation of *B*. *maydis* on leaves, and the changes remained in effect until at least 72 hpi. Several metabolic proteins in the leaves, stems, and roots of soybeans were invoked in response to *B*. *maydis*. Most of these proteins had essential functions, either in the regulation of the response, or in the metabolic process, allowing plants to survive and recover from the stresses [17].

The leaf protein RuBisCO operates as a metabolic branch point, channeling carbon either to the photosynthetic carbon fixation (Calvin–Benson) cycle or to the photorespiratory pathway. It is the most abundant protein in green plant tissues and is a bifunctional enzyme [28, 29]. Its most common form (type I presents in cyanobacteria, green algae, and higher plants) is composed of eight chloroplast-gene-encoded large subunits (~55 kDa) and eight nuclear gene-encoded small subunits (~15 kDa) [28, 29]. Two protein spots were identified as RuBisCO large subunits, which suggested that soybean photosynthesis was reprogrammed under *B*. *maydis* stress conditions.

BAHD acyltransferase catalyzes the acylation of various plant secondary metabolites [30]. The decrease in intensity of BAHD acyltransferase DCR-like (spot 66 in leaf) protein suggests that the metabolites were inhibited under *B*. *maydis* stress.

The intensity of the spot attributable to 4-hydroxy-3-methylbut-2-enyl diphosphate reductase (HDR) f-like protein decreased in response to *B*. *maydis*, which is also involved in a pivotal secondary metabolite process in NHR in soybeans (spot 77 in Table 1). Plant isoprenoids are involved in several key biological processes, including membrane fluidity, photosynthesis, growth regulation, cell division, communication, and defense response [31, 32]. Gene knockout has demonstrated that HDR activity is not only essential to *Escherichia coli* growth, but also plays a key role in plant isoprenoid biosynthesis [33–35]. Decreases in the intensity of leaf HDR and BAHD proteins suggests that *B*. *maydis* stress inhibited the metabolic processes of soybeans or some decrease in fitness is needed for the soybeans to resist *B*. *maydis*, although no visible symptoms have been detected.

There are many families of cysteine proteases in stems. These are also known as thiol proteases. Among these, C1A cysteine protease is the most abundant enzyme and is responsible for protein degradation during plant senescence [36]. Dozens of lattices from different plant families are known to contain cysteine proteases [37]. Several of these plant proteases accumulate in the central vacuole, from which these can be released as programmed cell death stimuli or other stress cues [25, 38]. These vacuole-localized proteases belong to the C1A family of cysteine proteases and are involved in both developmental and pathogen-related PCD [25]. Stem (spot 16) mitochondrial processing peptidase (MPP; EC 3.4.24.64) is a heterodimeric enzyme that plays a pivotal role in mitochondrial protein import. MPP is integrated into a protein complex of the respiratory chain in plants, and plays an essential role in proteolytical processing [39, 40]. The increase in the intensity of spots attributable to the production of subunits of MPP might be due to the NHR of soybean.

Heat shock proteins (Hsps) are molecular chaperones that facilitate proper protein folding and function in almost all organisms. These are classified into families based on their molecular weight: Hsps include Hsps 40, 60, 70, 90, and 100 [41]. In this present study, some Hsps were detected in either the chloroplasts or mitochondria. Stress often increases the number of unfolded proteins in the cytosol. The unfolded proteins require more molecular chaperones for re-folding, which makes the stressed cell more dependent upon the Hsps than unstressed cells [41]. In the present study, Hsp 70 was detected in stem protein 19, and it showed the most pronounced increase in spot intensity of any protein in the mitochondria (12-fold), which suggested that stem proteins were involved in the response to *B*. *maydis*.

The eukaryotic translation elongation factor plays an important role in translation elongation. The accumulation of maize chloroplast protein synthesis elongation factor has been reported to alleviate heat stress [42]. Elongation factor 1-delta-like protein was identified in root spot 1 (intensity increased by 2.7-fold) and elongation factor 1-gamma-like protein was found in root spot 19 (increased by 16.7-fold). Both were highly expressed in root cells, which suggested that the high level accumulation of elongation factor might be good evidence that new proteins are produced in soybean roots in response to exposure to the non-adapted pathogen *B*. *maydis*.

Protein spot 8 was attributed to root proteins related to increased protease concentration. This suggested that the protein degradation process also increased in response to *B*. *maydis* (Table 3), which suggested that protein processing occurred in the root organs of soybeans.

Some major metabolic processes such as photosynthesis, acylation of plant secondary metabolites, synthesis and degradation of proteins, and protein refolding, were determined to be involved in soybean NHR against *B*. *maydis*. Therefore, soybean plants can undergo NHR in response to resistant *B*. *maydis* at the expense of metabolism-related proteins.

**Fig 5:**
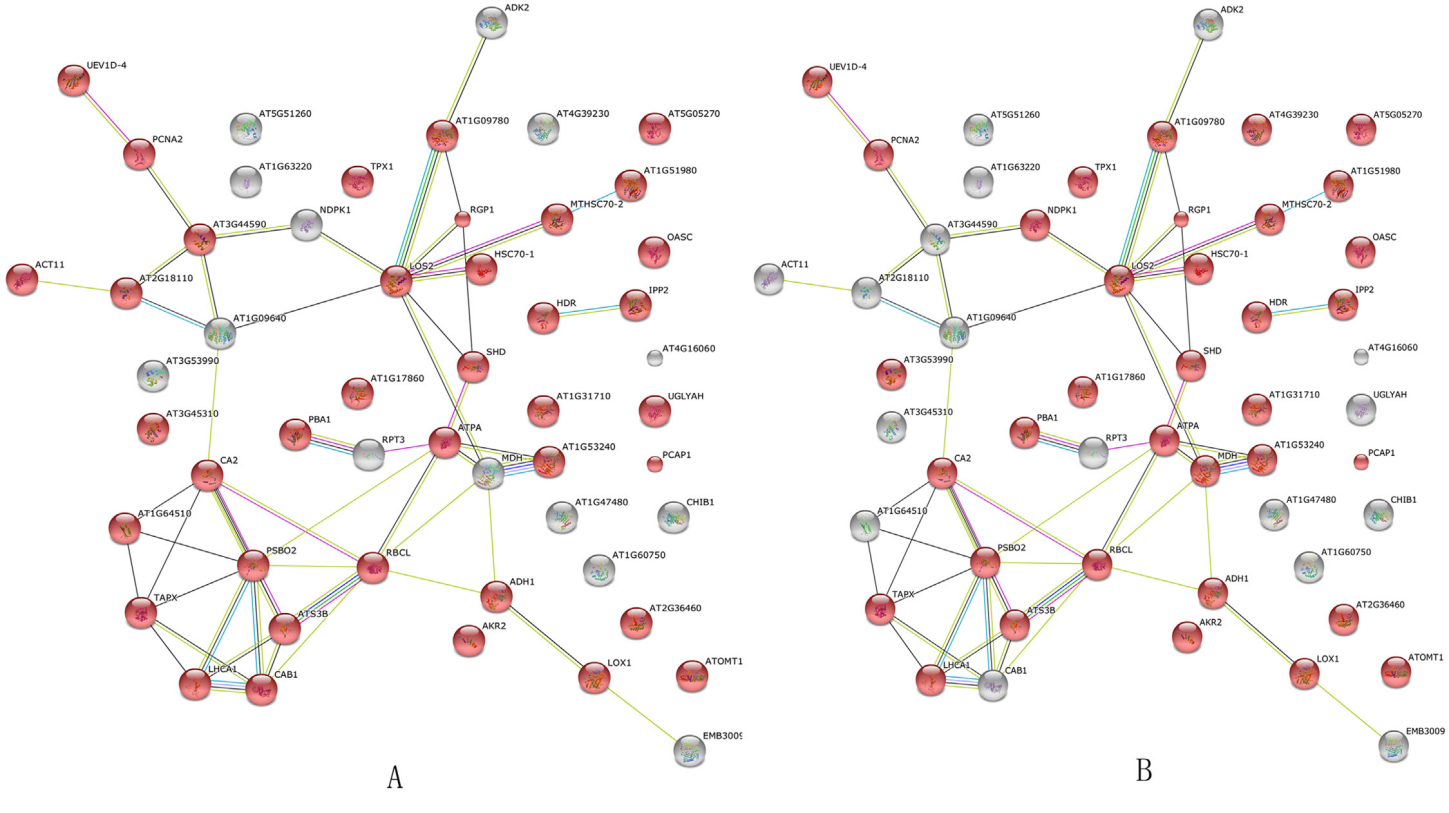
*Arabidopsis* DB-based BLAST analysis of the interaction network of 64 differentially expressed proteins in the roots, stems, and leaves of soybean plants in response to *B*. *maydis*. GO analysis indicated that the 38 red nodes in A might be involved in metabolic processes (P value = 1.070e ^−10^). Another 35 red nodes in B were involved in stimulus response (P value = 3.970e ^−10^). Thirty proteins belonged to the both metabolic process and response to stimulus, which suggested that the same protein could perform different functions in soybean plants in response to *B*. *maydis* stress.

Analysis of the interactions among 64 proteins differentially expressed in the roots, stems, and leaves of soybeans in response to *B*. *maydis* provided further bioinformatic evidence of the overlap between metabolism and stimulus response. This analysis was performed using STRING (Fig 5). The results suggested that ~80% of proteins were involved in metabolic processes (*P* = 1.070e -10) and response to stimulus (*P* = 3.970e -10). For example, LOS2 is a copper ion binding/phosphopyruvate hydratase. It encodes an enolase that is involved in light-dependent cold tolerance. Its protein is tyrosine-phosphorylated and its phosphorylation state is modulated in response to ABA in *Arabidopsis thaliana* seeds (www.string.db.org) (Fig 5). BLAST analysis using the *Arabidopsis* DB was performed. These proteins belonged to both the response pathway and biological processes. This theoretical overlap of metabolism and defense processes was consistent with the observed overlap between metabolic and defense processes (Fig 4, Tables 1–3).

### Defense-related proteins in response to *B*. *maydis*

In addition to co-induction of metabolic proteins against *B*. *maydis*, NHR in soybeans also activates defense-related proteins in response to *B*. *maydis*. Chitinases expressed during plant-pathogen interactions are associated with plant defense against pathogens, which can catalyze polychitin molecules that are present in the cell walls of most fungi and homologues in plant-pathogen interactions [43]. Plants possess a large reservoir of protease inhibitor (PI) genes, which have been proposed to act as storage proteins, regulators of endogenous proteases, and most notably in defense against herbivores. They have potent applications in resistant crop breeding [44]. However, an Ecc licitor-induced Kunitz trypsin inhibitor gene (AtKTI1) encoding a functional Kunitz type PI protein was isolated in *Arabidopsis*, and demonstrated that the PI protein played a regulatory role in PCD antagonizing pathogen and FB1-induced cell death [44]. In the present study, the expression of the trypsin inhibitor and acidic chitinase increased in the vacuoles of soybean leaf cells, which indicated that these played key roles in NHR of soybeans to *B*. *maydis*.

Malate dehydrogenases (MDHs) often use malate or oxaloacetate as substrates to generate pyruvate and preferentially utilize NADP or NAD as cofactors. These differ in their subcellular localization among and within species [45]. The function and regulation of MDH in animals are specifically associated with cell differentiation and proliferation, ontogenic development, hormonal control, and diseases [45]. Stem protein spots 23 and 24 were identified as malate dehydrogenases (MDHs), which suggested that these might play a role in soybean NHR against *B*. *maydis* and provide new insights into their regulatory function beyond their classical role in basic metabolism.

Isoflavone reductase is an enzyme associated with isoflavonoid biosynthesis in plants, and its overexpression of rice isoflavone reductase-like gene (OsIRL) causes tolerance to reactive oxygen species, thus indicating that rice isoflavone reductase plays an essential role in maintaining the levels of ROS [46]. Root protein spot 16 was identified as soybean isoflavone reductase-like protein, which might be involved in NHR of soybean to *B*. *maydis*.

Cell-wall-associated kinases (WAKs 1–5) and their isoforms have been shown to functionally mediate differential signals from the extracellular matrix to the cells [47]. Oxygen evolving enhancer protein 1 (OEE1) is a chloroplast protein that acts as an auxiliary component of the photosystem II manganese cluster. A previous study has shown that OEE2 interacts with and acts as a substrate for WAK1, and is phosphorylated via the action of AtGRP-3 [47]. Phosphorylation of OEE2 is also induced in *Arabidopsis* by exposure to the avirulent *Pseudomonas syringae*. OEE2 is downstream of *AtGRP-3/WAK1*, and may be involved in defense signaling [47, 48]. OEE1 was upregulated 11-fold in this present study (stem spot 7 and upregulated 2.5-fold in leaf protein spot 34), which suggested that strong defense signaling had been taking place in the soybean cells under the *B*. *maydis* stress conditions. In addition, Ning’s results showed that OEE played an important role in photosynthesis and stress resistance for plants [49]. Its subcellular location was predicted in chloroplasts.

### Similarity and differences of NHR in soybean and other plants

The proteomic basis of NHR in the soybean response to *B*. *maydis*, a pathogen affecting remotely related species, was assessed. Results showed the soybean NHR response to have many similarities to that of other plants such as callose deposition and H_2_O_2_ production [9]. The basic biological processes involved in soybean NHR against *B*. *maydis*, including some vital metabolic components for plant growth and development such as RuBisCO, BAHD, and OEE were different from the NHRs of other plants. Soybean NHR involved almost all of the organelle (Fig 4, Table 4); this is the first work to indicate the involvement of NHR in organelles.

**Table 4:** Predicted locations of differentially expressed proteins in soybean to *B*. *maydis*.

Organ	Predicted location								
	Chloroplast	Cytoplasm	Nucleus	Vacuole	Endoplasmic reticulum	Golgi apparatus	Mitochondrion	Cell wall	Cell membrane
Leaf	8	4	1	2	0	0	0	0	0
Stem	7*	3	2	1	1	1	6	0	0
Root	5*	9	3	0	0	1	1	1	1

Some proteins are predicted in multiple locations, which could be located in chloroplast and mitochondrion.

These results have allowed us to propose a hypothesis relating to soybean NHR, which affected almost all of the organelles in the cells of soybean plants (Fig 4 and Table 4). Soybean NHR against *B*. *maydis* is a type I nonhost resistance. Unlike host resistance that is mediated by the products of plant resistance genes (R), which involves pathogen-race- or plant-cultivar-specific interaction [9], the soybean NHR involved multiple-gene resistance, which involved multiple biological process, cellular components, and molecular functions in response to non-adapted pathogens. This may explain why a pathogen that is virulent to one plant species is nonpathogenic to another. These results could not only be used in intercropping systems involving soybeans but also in rotations involving nonhost plants in an agroecosystem.

A preliminary conclusion can be drawn from the results of the present study. Soybeans can respond to *B*. *maydis* stimulus and this involves almost all subcellular structures and major metabolic processes and multiple-gene resistance through reprogramming far from the site of inoculation. This response included multiple biological processes, cellular components, and molecular functions. Some major proteins such as RuBisCO, BAHD, NDK, and OEE were involved in NHR in soybean and overlapped with metabolic proteins. Proteins related to metabolic processes were co-induced in response to non-adapted pathogens, which differs from the results of previous studies of NHR [1–3; 8–10; 12–13]. Similarly, the expression of acidic chitinases, protease, and Kunitz type PI (protease inhibitor) proteins changed in the vacuoles of soybean leaf cells, which indicated that these played major roles in NHR of soybeans to *B*. *maydis*. This is the first report to find evidence for a multiple-responsive NHR in soybeans against *B*. *maydis*, a pathogenic fungus of a phylogenetically distant crop. These findings may offer insights into the microbe-mediated interactions between plant species, which play a basic role in shaping natural communities and crop yields [26, 27]. However, identification of the associations among the systematic responses and assessment of resistance at multiple levels in soybeans require further investigation.

## Supporting Information

S1 Fig Cytological stress response indicated via H_2_O_2_ production. H_2_O_2_ *in situ* leaves of soybean seedlings exposed to *B*. *maydis*, DAB was allowed to react with H_2_O_2_, producing a brown polymerization product in the presence of peroxidases. Hyphae (HY) germinated from the conidia (CO) might be delimited by H_2_O_2_ production. The color mainly appeared oriented to the position localized by the interaction between soybean leaves and *B*. *maydis*. Bars are 20 μm in A and B. The microscope and software used for image handling were LEICA DM2000 and Adobe Photoshop 7.0.1.
(TIF)

S2 Fig 2-DE map of control soybean stem proteins. (TIF)

S3 Fig 2-DE map of treated soybean stem proteins. (TIF)

S4 Fig 2-DE map of control soybean leaf proteins. (TIF)

S5 Fig 2-DE map of soybean leaf proteins. (TIF)
