# Supplementary figures and images for: Proteomic Analysis of the Relationship between Metabolism and Nonhost Resistance in Soybean Exposed to Bipolaris maydis
Source: PLoS One. 2015 Oct 29;10(10):e0141264. doi: 10.1371/journal.pone.0141264 (PMC4626022; doi:10.1371/journal.pone.0141264)

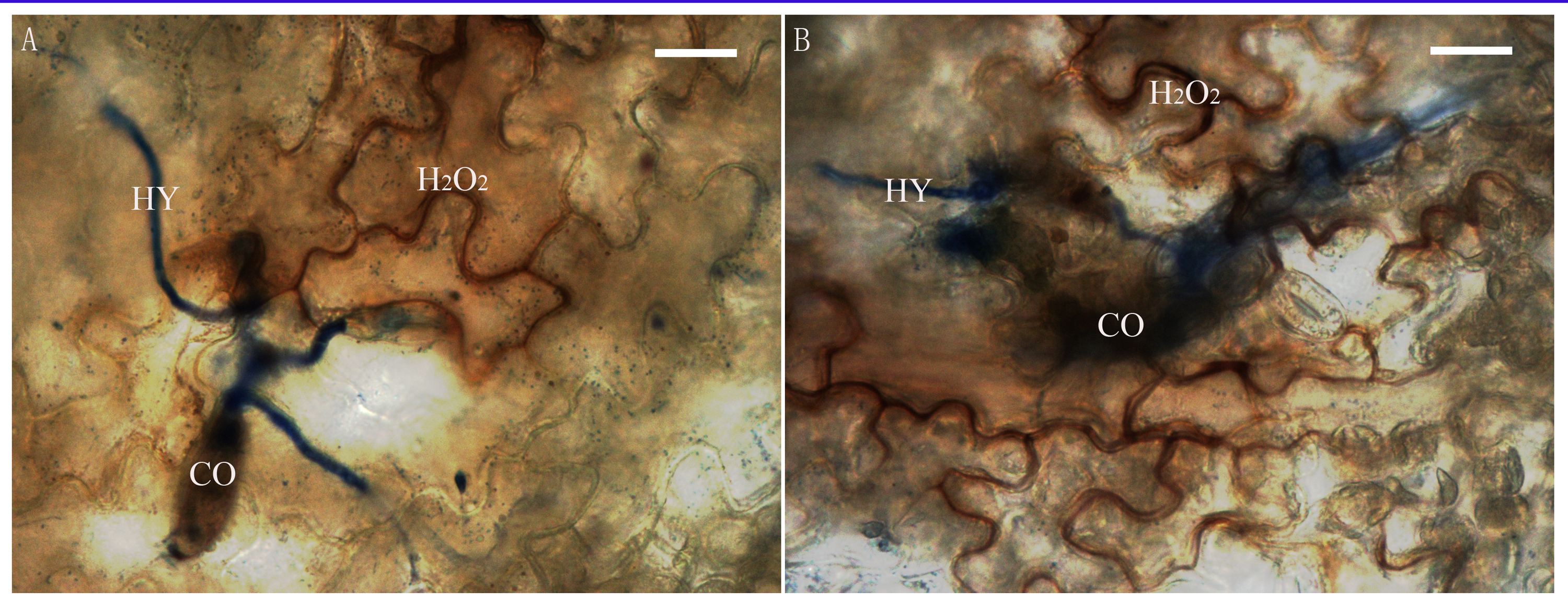

Supplement: S1 Fig — H2O2 in situ leaves of soybean seedlings exposed to B. maydis, DAB was allowed to react with H2O2, producing a brown polymerization product in the presence of peroxidases. Hyphae (HY) germinated from the conidia (CO) might be delimited by H2O2 production. The color mainly appeared oriented to the position localized by the interaction between soybean leaves and B. maydis. Bars are 20 μm in A and B. The microscope and software used for image handling were LEICA DM2000 and Adobe Photoshop 7.0.1. (TIF) [file pone.0141264.s001.tif]

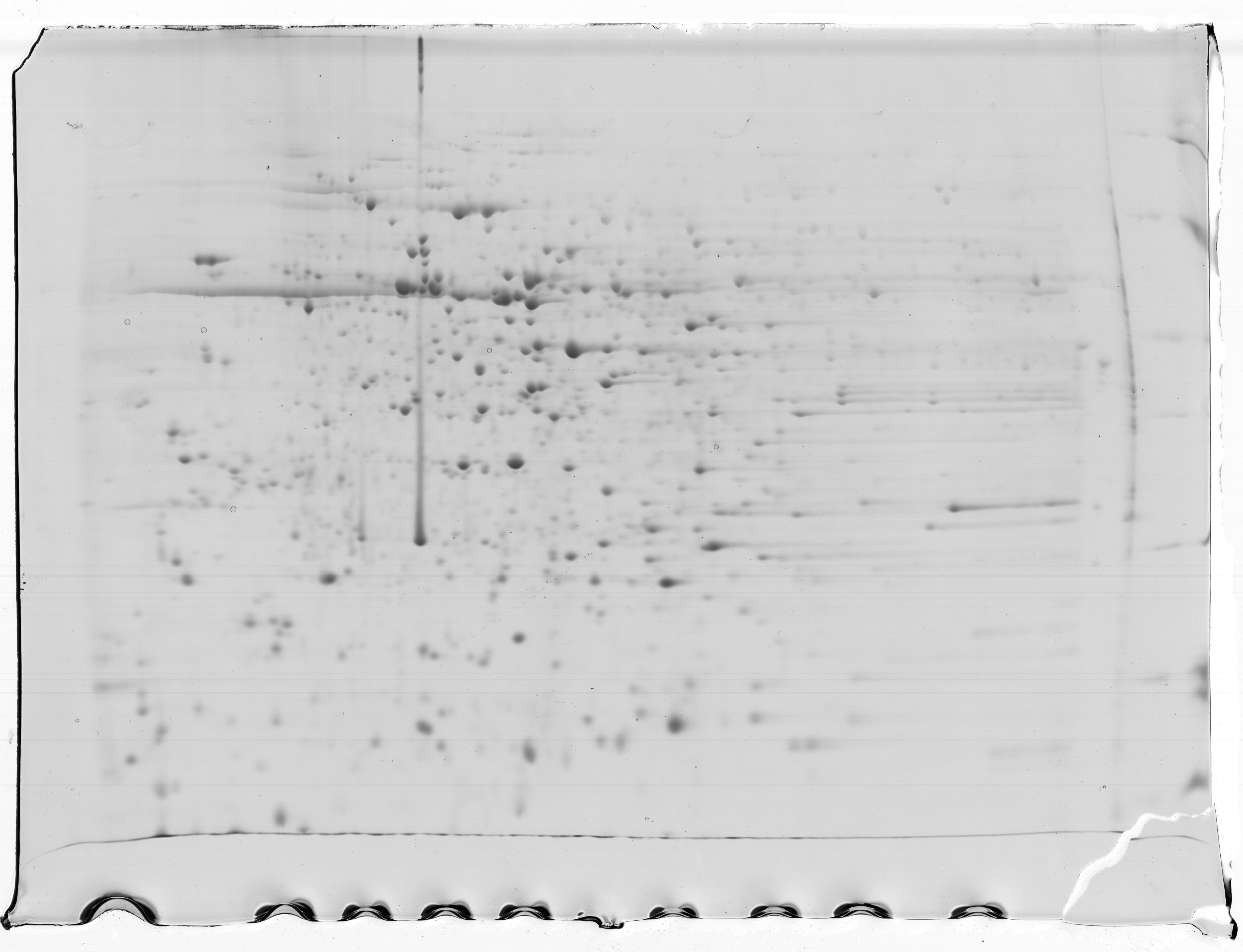

Supplement: S2 Fig — (TIF) [file pone.0141264.s002.tif]

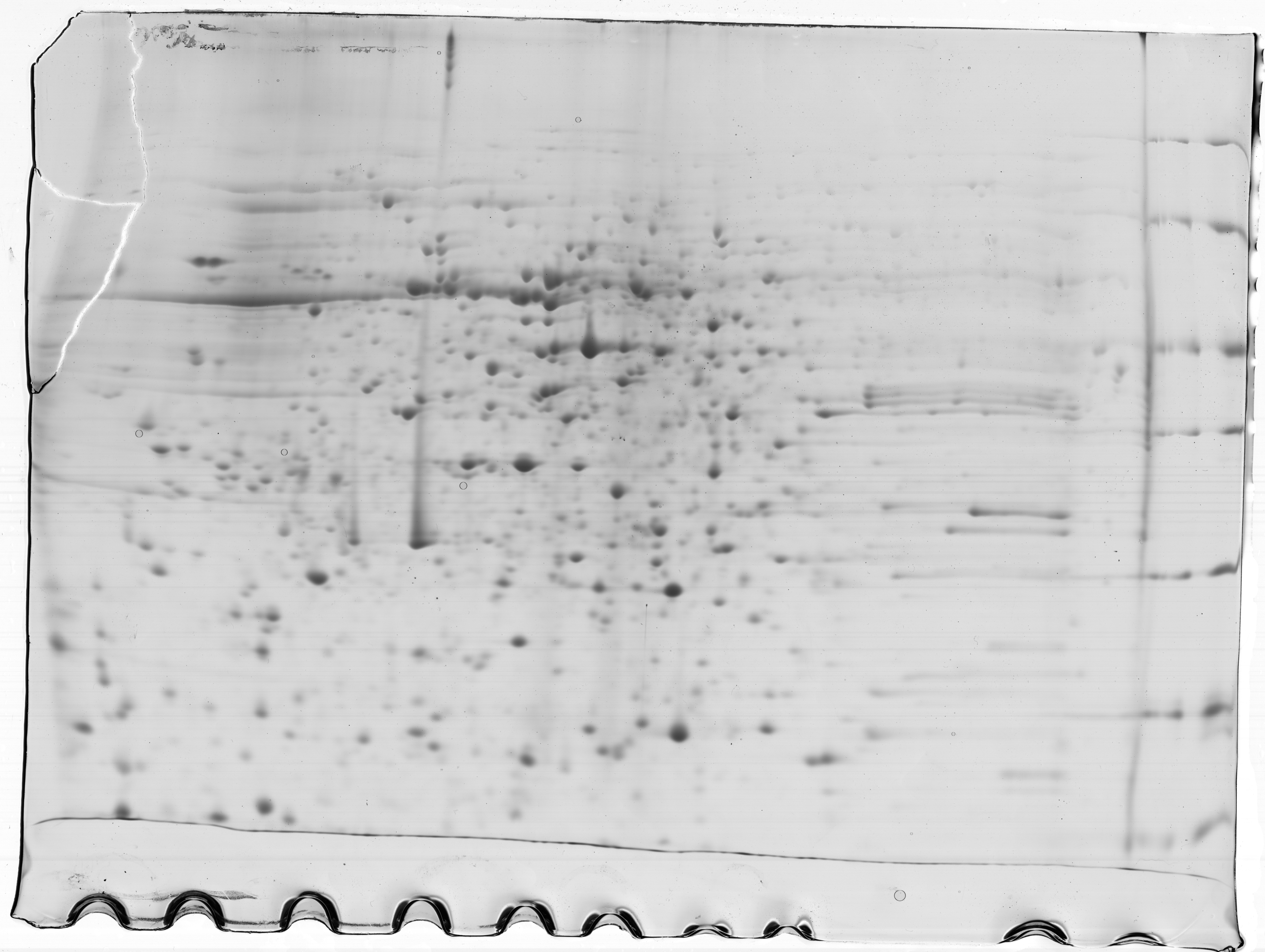

Supplement: S3 Fig — (TIF) [file pone.0141264.s003.tif]

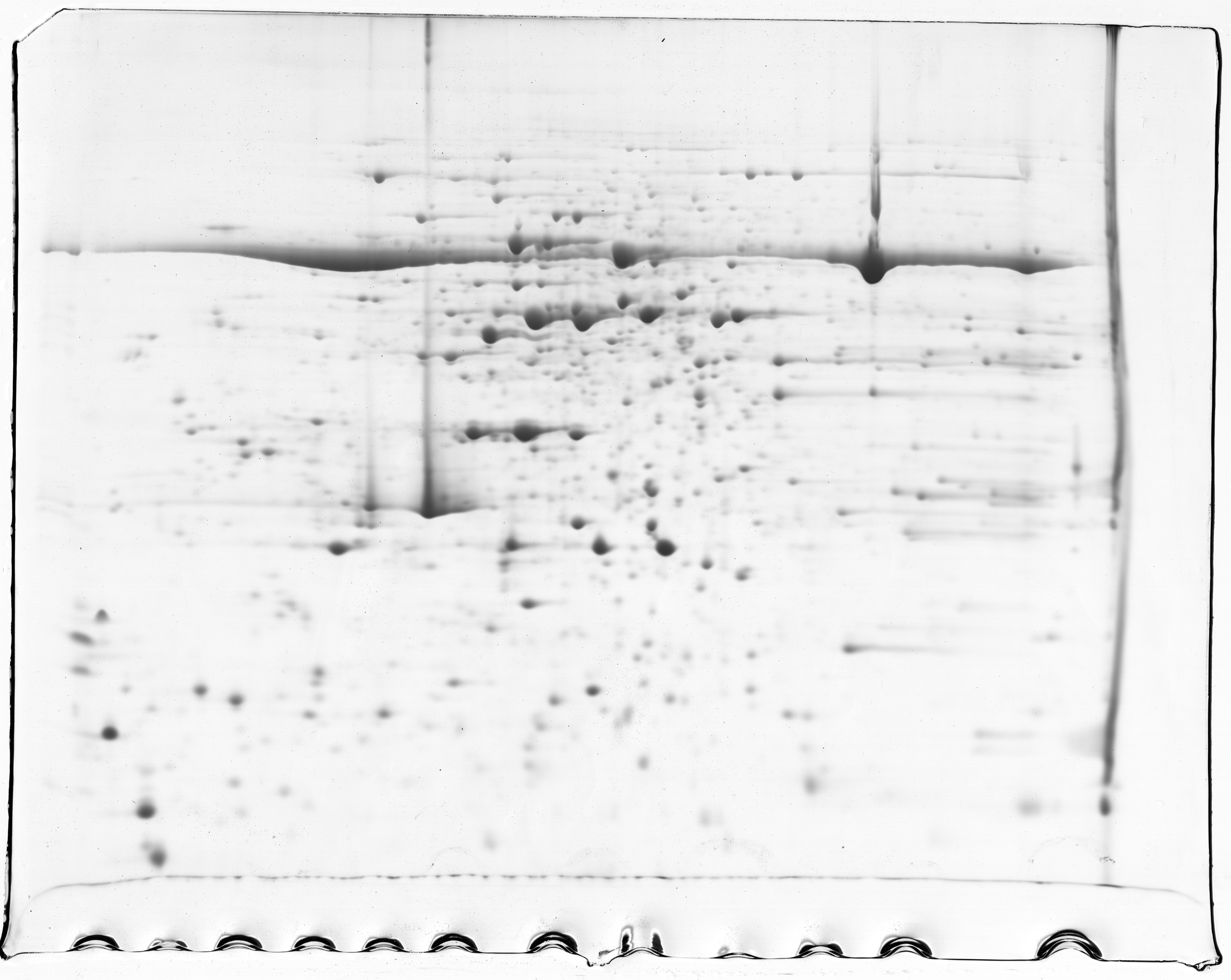

Supplement: S4 Fig — (TIF) [file pone.0141264.s004.tif]

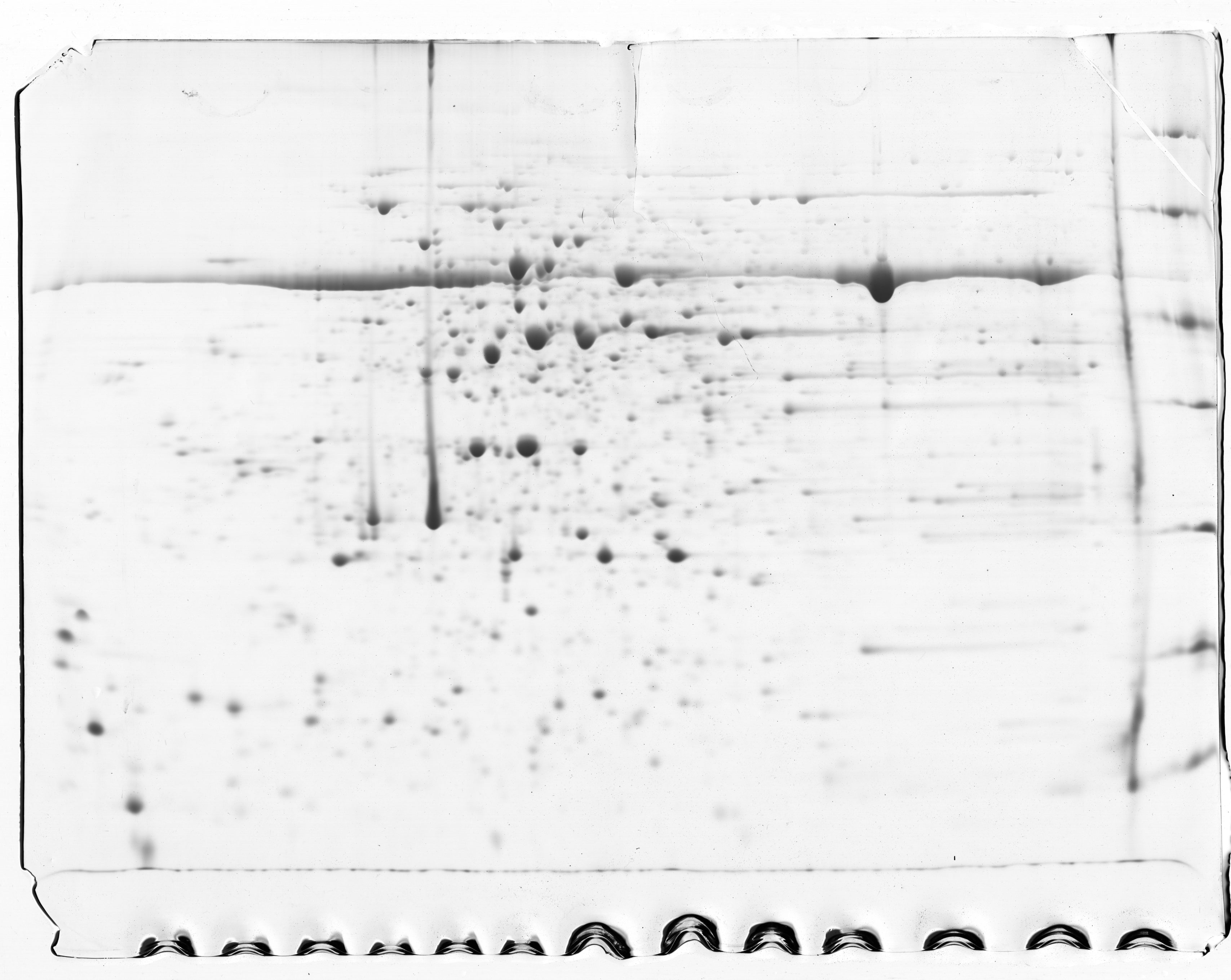

Supplement: S5 Fig — (TIF) [file pone.0141264.s005.tif]
